# Quantifying effects of the European Health Data Space on the app ecosystem and data access

**DOI:** 10.1038/s41746-026-02917-7

**Published:** 2026-06-19

**Authors:** Stefanie Brückner, Ronja Riedel, Sven Hetmank, Kyra Fritsch, Oscar Freyer, Stephen Gilbert

**Affiliations:** 1 Else Kröner Fresenius Center for Digital Health, Technische Universität Dresden, Dresden, Germany; 2 Institute of International Law, Intellectual Property and Technology Law, TUD Dresden University of Technology, Dresden, Germany

**Keywords:** Health care, Mathematics and computing

## Abstract

Access to patient-generated health data from mobile apps and wearables is increasingly central to connected care. The European Health Data Space (EHDS) introduces app providers as health data holders with obligations to share data for patient care and secondary use. We mapped EHDS health data holder criteria and applied them to a systematic review of 100 health apps, using privacy policies and related public sources to identify disclosed health data types and processing purposes. Overall, 18% of apps could qualify under the Personal Health Data Pathway and 21% under the Anonymous Health Data Pathway. Extrapolated to the currently globally available 337,000 health apps in app stores, more than 60,700 and 70,800 app providers could qualify. This suggests that a substantial share of consumer health apps may become integrated into the regulated EHDS secondary-use ecosystem. The scale of data availability will depend on enforcement and how providers adapt their data practices.

## Introduction

The European Health Data Space Regulation (EHDS-R) establishes a harmonised EU-wide framework for the access, use, and exchange of electronic health data across Member States^1^. For health apps, it sets out how app-derived health data can be incorporated into electronic health records for direct care (primary use) and the conditions under which app providers must make health data available for secondary uses, such as medical research, public health, and policy development^2^. The EHDS-R was adopted in February 2025 and entered into force in March 2025. It will apply in phases from March 2027, with the secondary use framework relevant to health data holders applying from March 2029. The regulation reflects the growing role of digital technologies in healthcare, where self-care often begins at home. Health apps now routinely collect vast amounts of sensitive data, including cycle diaries or heart rate measurements. This valuable patient-generated health data (PGHD) can be used, for example, for menstrual and reproductive health monitoring^3^, and beyond personal care, for population-level infectious disease surveillance^4^.

Under the EHDS-R, health apps are grouped by their function and context of use into two categories. For the first time, the regulation provides a legal definition of a *wellness application*: software that processes electronic health data to provide information on an individual’s health or to support care, while excluding the provision of healthcare (Art. 2(2)(ab) EHDS-R). *Healthcare* is defined as services provided by health professionals to patients (Art. 2(1)(b) EHDS-R) and Art. 3(a), Directive 2011/24/EU)^5^. Wellness applications, therefore, operate outside professional care settings. The second group comprises products and services intended for the health, healthcare, and care sector. This grouping is independent of the app’s medical device (MD) status under the Medical Device Regulation (MDR)^6^.

This study examines the roles of app providers as data holders under the EHDS-R and their obligations to make this data available for secondary use to leverage the full potential of digital technologies beyond consumer self-care. It combines a regulatory analysis of the EHDS-R with an empirical review of 100 health apps and their publicly disclosed data-sharing practices, drawing on Privacy Policies, app store listings, and product websites. The study provides insights into how many apps are affected and the practical implications for providers and for access to app-generated data within the EHDS. Throughout this study, we use the term app provider to refer to the entity that makes the app available to users and determines the purposes and means of processing app-generated data. This may differ from the software developer in a technical sense. Although the EHDS-R refers to entities “developing” relevant products, services or wellness applications (Art. 2(2)(t) EHDS-R), the obligations analysed here concern the entity that controls the app service, sets the privacy policy, and would be expected to respond to health data access requests.

## Results

### The route for app providers to become an EHDS-R health data holder

**Fig. 1:**
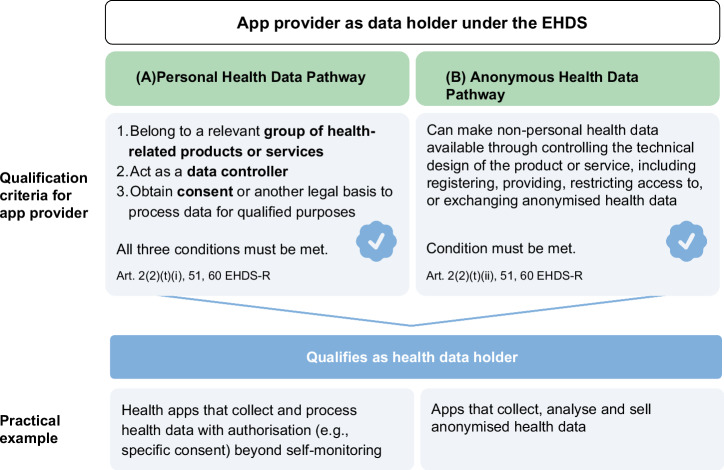
EHDS health data holder pathways for app providers. Overview of pathways and practical examples of how health data from health apps might be made available for secondary use under the EHDS Regulation.

The regulatory analysis of the EHDS-R identified two possible pathways through which app providers may qualify as data holders under the EHDS-R: the Personal Health Data Pathway and the Anonymous Health Data Pathway. Figure 1 summarises the qualification criteria and provides practical examples for each pathway.

### Empirical analysis of health apps to identify potential data holders under the EHDS Regulation

**Fig. 2:**
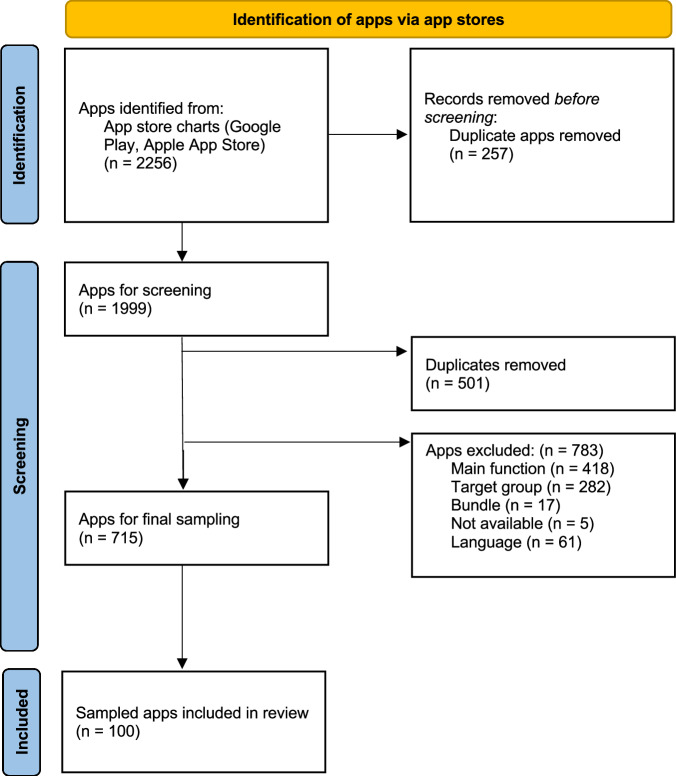
App identification, screening, and sampling workflow. Records from Google Play and Apple App Store charts were de-duplicated, screened against eligibility criteria, and sampled to obtain the final 100-app review set.

Results of the app identification and sampling process are summarised in Fig. 2. From 2256 apps retrieved from the app charts, 715 eligible apps across 15 function groups remained after duplicate removal and eligibility screening. The final assessment sample comprised 100 apps, including 15 medical device apps and 85 non-medical-device apps, with function groups proportionally reflecting the distribution in the eligible app dataset (Table 1).

**Table 1:** List of apps classified based on their primary function and sampled numbers

Function groups	Number of apps	% of total	Sampled app numbers	
Fitness, Activity & Workout	208	29.1	23	↓
Disease Management & Therapy	111	15.5	19	↑
Nutrition & Calorie Counter	67	9.4	9	=
Sexual & Reproductive Health	64	9.0	9	=
Physiological Measurements	58	8.1	8	=
Mental Health & Wellbeing	39	5.5	6	=
ePharmacy & ePrescription	38	5.3	4	↓
Sleep Tracker	30	4.2	5	↑
HCP Access & Communication	21	2.9	3	=
Speech & Cognitive Training	20	2.8	3	=
Health Insurance Management	16	2.2	2	=
Diagnosis-support	15	2.1	2	=
PHR & Patient Portal	15	2.1	5	↑
Device Control	7	1.0	1	=
Hygiene, Cosmetic & Homoeopathy	6	0.8	1	=
Total	715	100	100	

Stratified function-group sampling is indicated: oversampled (↑), undersampled (↓) and proportional (=). *HCP* Healthcare Professional, *PHR* Patient Health Record.

Table 2 presents the resulting app qualification outcomes under the Personal and Anonymous Health Data Pathways of the EHDS-R, stratified by medical device status. Personal health data included any app-generated data that could directly or indirectly reveal a user’s health status, including pseudonymised data, while anonymous health data referred to non-personal health data described by the app provider as anonymised or otherwise no longer linked to an identifiable user. Classifications reflect the data practices disclosed in privacy policies. Actual data flows, app provider access to personal data, anonymisation methods, or residual re-identification risk were not verified. The complete data extraction file is available in Supplementary Table 2. Of the 100 apps, 20 stored health data solely on the user’s device, thereby excluding the app provider from qualification as a data holder.

**Table 2:** App qualification as EHDS health data holders

Pathway	Evaluation	Total apps	MD	Non-MD
(A) Personal Health Data Pathway	Yes	18	11	7
	Unclear	4	1	3
	No	78	3	75
(B) Anonymous Health Data Pathway	Yes	21	7	14
	Unclear	12	2	10
	No	67	6	61

Qualification outcomes for the Personal Health Data Pathway and Anonymous Health Data Pathway are stratified by medical device status.

*MD* Medical Device.

For the Personal Health Data Pathway, 18 of 100 health apps fulfil all three criteria (Table 2A). Among these, MD apps dominate (11/18) with five (5/11) mentioning health data processing for healthcare, three (3/11) for research and three (3/11) covering both purposes. Of the remaining seven non-MD apps (7/18), two list healthcare (2/7), one lists healthcare and research (1/7), and four list research as their health-data processing purposes (4/7). Most of the apps (11/18) that qualify as data holders in this pathway belong to the Disease Management & Therapy app group. Supplementary Table 3 shows the detailed analysis of the app function group and data holder status.

Four apps (4/100) were rated as unclear for the Personal Health Data Pathway. Of these, three apps (3/4) mention research as a purpose for health data processing, but their privacy policies did not sufficiently describe the nature of the research for a final assessment. It may be scientific research or only performance analytics. Two of these apps (2/3) feature publications on their website, making a qualification as a health data holder more likely. The fourth app in this group stated that it uses user health data to train AI, which may constitute innovation.

The sample was further analysed for the edge-case health data processing purposes analytics and market research. Fifteen apps (15/100) mentioned analytics with health data or general personal data that could include health information. Of these, six (6/100) already fulfilled all criteria for the Pseudonymised Pathway, and one (1/15) was unclear (innovation purpose). The remaining eight (8/15) could qualify if analytics were treated as a qualifying purpose. Market research was mentioned in Privacy Policies only in connection with app-external data collection activities, such as video interviews or email surveys, that required additional consent and were therefore not further analysed.

A special subset of apps (3/100) in our sample consists of two health insurance management apps offered by statutory health insurers for submitting reimbursement claims and managing insurance-related matters, and one electronic patient record from a statutory health insurer. Under the EHDS, health insurance providers are entities in the healthcare sector authorised to process personal electronic health data for purposes including reimbursement, patient safety, and official statistics, and are therefore health data holders. However, statutory insurers occupy a distinct regulated role that differs from private app providers and was not the focus of the app-provider pathway analysis. These apps were therefore retained in the sample but were not counted as qualifying app-provider data holders under either the Personal or Anonymous Health Data Pathway.

The majority of apps (78/100 apps with 3 MD apps) did not qualify as health data holders through the Personal Health Data Pathway.

### Anonymous Health Data Pathway qualification for health apps

Table 2B provides an overview of the results from the Anonymous Health Data Pathway analysis of the app sample. In our sample, 21 apps (21/100) qualify as data holders. A further 12 (12/100) received an unclear rating due to missing details in the privacy policy: Seven (7/12) did not specify whether user data also contains health data or only usage data, three (3/12) declared any processing of anonymous data as out of scope of privacy policy, and one (1/12) used anonymous AI chatbot data where it is not clear that it contains health data. One (1/12) app reports processing aggregated data, but does not clarify whether the data are also anonymised. Supplementary Table 3 shows the detailed analysis of the app function group and data holder status for this pathway. A total of twelve apps (12/100) qualified as data holders for the Personal and the Anonymous Health Data Pathway.

### Estimated global relevance

**Fig. 3:**
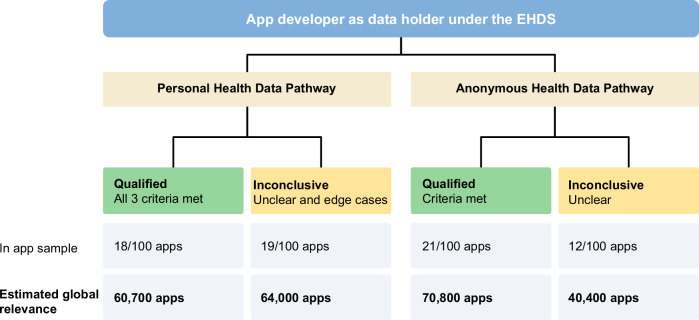
Estimated global relevance of EHDS health data holder pathways for health apps. Overview summarises pathway qualification in the 100-app sample and extrapolates the observed proportions to a global estimate of 337,000 health apps available in app stores.

In summary, 18 apps (18/100) in our sample qualified directly as health data holders via the Personal Health Data Pathway and 21 (21/100) via the Anonymised Health Data Pathway; 12% met both. To estimate the possible scale, we applied the pathway-specific proportions observed in the 100-app sample to external global health app market estimates^7^. In the sample, 18% of apps qualified via the Personal Health Data Pathway and 21% via the Anonymous Health Data Pathway, with 12% qualifying through both pathways. Applied to the global estimate of 337,000 health apps available in app stores, this exploratory scenario suggests that approximately 60,700 apps could qualify as data holders via the Personal Health Data Pathway and 70,800 via the Anonymous Health Data Pathway. Approximately 40,400 apps could qualify through both pathways (Fig. 3). As an additional scenario estimate, we applied the same proportions to UK health app statistics for a market of comparable size to the EU^8^. Based on 227,500 health apps reported as available in the UK, this would correspond to approximately 41,000 app providers via the Personal Health Data Pathway and 47,800 via the Anonymous Health Data Pathway, with around 27,300 potentially qualifying through both pathways.

## Discussion

This study shows that app providers may qualify as EHDS health data holders through both personal and anonymous health-data routes, but that qualification is often difficult to determine from publicly available information.

In the app sample, 18 of 100 apps met all criteria for the Personal Health Data Pathway. The evaluation was challenged by the undefined terms of research and innovation, as well as vaguely written Privacy Policies. The newly released EDPB Guidelines on processing of personal data for scientific research purposes provide useful interpretative context: scientific research should be understood broadly, including privately funded research and technological development, but only where the activity is genuinely scientific and supported by factors such as a methodical approach, ethical standards, transparency, researcher independence, and contribution to general knowledge^9^. Hence, vague privacy-policy references to “research” are insufficient to determine whether the processing would qualify as scientific research rather than product usability testing, internal analytics, or commercial optimisation. In our sample, four apps were therefore classified as unclear for the Personal Health Data Pathway, and 15 apps were flagged as edge cases.

For the Anonymous Health Data Pathway, 21 of the 100 apps in our sample met these criteria. While privacy policies must state that they anonymise personal data for further processing and use, as required under the GDPR, they do not have to specify exactly what they do with this data^10^. Consequently, some Privacy Policies were very transparent about how they use anonymised user data, e.g., for research collaborations with partners. In contrast, others simply stated that this was out of scope for this policy.

During the development and passage of the EHDS-R, the territorial scope of data holder obligations for app providers was subject of uncertainty, as reflected in changes between earlier draft versions and the final regulation^1,11^. Further clarification was provided in the European Commission’s explanatory FAQ on the EHDS, published in March 2026, which states that EHDS obligations apply to data holders established in the EU and do not apply to data holders established in non-EU countries unless they have an established presence in the EU (response to Question 56)^12^. However, whether a health app provider has a legal or physical establishment in the EU cannot be reliably determined from app store listings or publicly available company information, as it may be a subsidiary of a primary establishment elsewhere. For this reason, the territorial establishment of the health app provider was not used to determine if they qualify as a data holder.

The central question is to what extent the EHDS-R will increase the secondary use of health app data. To illustrate the possible scale of this effect, we applied proportions observed in our EU-facing app sample to external global app market estimates. This exploratory scenario suggests that approximately 60,700 and 70,800 apps could become data holders via the Personal or the Anonymous Health Data Pathway (see Fig. 3). A total of 40,400 could qualify through both pathways. As a comparator, applying the same proportions to available UK health app statistics would correspond to approximately 41,000 and 47,800 app providers via the Personal and Anonymous Health Data Pathways, respectively^8^. These figures should be interpreted as order-of-magnitude estimates rather than precise market counts, as no EU-wide health app statistics were available and the app store sample was designed to capture visible health apps in selected EU markets rather than to provide a statistically representative EU-wide sample. It remains unclear how data holder requirements, including territorial scope as clarified through further interpretation and case law, will shape app business practices. Some companies may, for example, choose business models that avoid establishing in the Union, although making their app available to the Union population. The influence of the Brussels Effect is also unknown, i.e., where entities end up complying with Union laws even when outside the Union, and even when not formally legally bound by these laws^13^. The numbers of app provider potentially considered a health data holder should therefore only serve as an indication of the wider app ecosystem that could be affected.

**Fig. 4:**
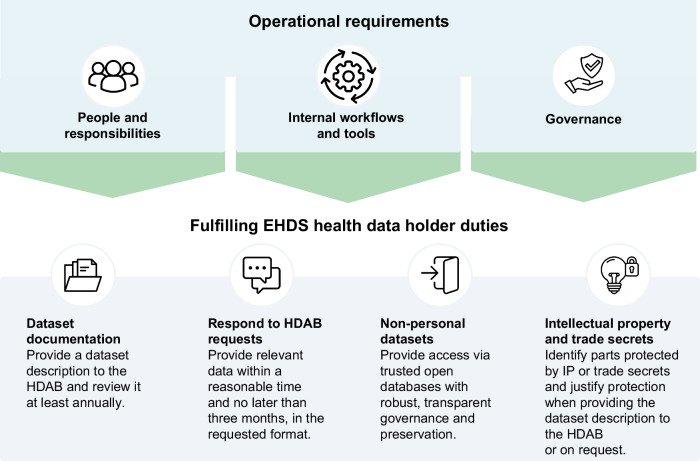
Operational requirements for the app provider to fulfil EHDS health data holder duties. Providers need staff, responsibilities, workflows, and governance for dataset documentation, HDAB requests, non-personal data access, and protected assets. HDAB Health Data Access Body, IP Intellectual property.

The designation of app providers as EHDS health data holders fundamentally reshapes their roles. They no longer operate solely as consumer-facing service providers but as regulated actors within the European health data infrastructure. Providers will need internal processes to receive and respond to HDAB requests within the specified timelines (Art. 68(7) EHDS-R) and to maintain transparent dataset documentation with up-to-date descriptions (Art. 60(3) EHDS-R) (Fig. 4). Safeguards for intellectual property and commercially sensitive information apply, but do not justify refusing access 52 EHDS-R). For non-personal data holders, obligations are even more active: data should be made available via trusted open databases to enable unrestricted use and preservation (Art. 60(5) EHDS-R).

The practical impact of the EHDS will also depend on its phased implementation timeline. The regulation entered into force in March 2025 and generally applies from March 2027, when several implementing acts are expected to define key technical, organisational, and procedural requirements. The secondary use framework, including the main obligations for health data holders, applies from March 2029. App providers that qualify as health data holders may therefore need to assess before this date whether they hold relevant electronic health data, including historic data collected before 2029, and whether their governance, documentation, and technical systems can support data availability through national health data access bodies (HDAB).

Ultimately, the real-world impact of the EHDS-R on the secondary use of app-generated health data will depend on how providers respond to its obligations. Many apps currently rely on broad consent without specific plans for research or innovation. Once providers recognise that such a legal basis could trigger EHDS health data holder duties under the Personal Health Data Pathway, some may narrow their approach or choose not to seek such consent to avoid obligations. Non-personal health data holders could also expect substantial consequences. Commercial apps often monetise access to anonymised user data by selling it to third parties.

Some app providers may perceive EHDS data sharing obligations as commercially sensitive, particularly when curated datasets are part of their data assets. However, the EHDS-R does not provide unrestricted access to such data. Secondary use is limited to permitted purposes, subject to authorisation by HDAB, data minimisation, secure processing environments, prohibitions on re-identification and prohibited uses, and safeguards for intellectual property rights and trade secrets (Art. 52 EHDS-R). Therefore, the commercial impact on app providers will depend on how these safeguards are implemented and how data access requests involving commercially sensitive datasets are handled in practice.

Lastly, the EHDS-R introduces a legal definition of *wellness application* based on an app’s health data processing function and context of use, which is not linked to the conventional use of this term for general wellbeing and lifestyle applications^14^, to separate them from formally approved MD (under the MDR). To qualify as a health data holder for secondary use, the MD status of a health app is irrelevant as long as it belongs to the relevant group of (i) products and services intended for the health/healthcare/care sector or (ii) wellness applications. One can argue that, although not named separately, MDs fall into the first group. The complication is that many MD apps do not meet the definition of *healthcare* or *care* provided in the EHDS-R, as they are used without clinicians or care institutions in the loop. Hence, a single app can be an MDR MD and an EHDS-R wellness application at the same time. This is not a problem for secondary data use; however, the situation is different for primary data use. Here, MDs and wellness applications have different requirements to participate in data sharing with EHR systems (Table 3). To demonstrate the confusion an app provider might face: consider a class I menstrual cycle app that is an MD but is used entirely self-directedly. Functionally, it may fall under the EHDS-R definition of wellness application, yet, for primary use data sharing, should it undergo the wellness application route or the MD route? Without guidance, providers face uncertainty about which obligations apply in which context. This confusion would have been preventable if the legislator had provided a more precise definition of wellness application or clearer guidance on how the MDR status of a health app impacts certain obligations.

**Table 3:** Pathways for secondary use of app-generated health data under the EHDS Regulation

	(A) Personal Health Data Pathway	(B) Anonymous Health Data Pathway	(C) EHR Interoperability Health Data Pathway
EHDS-R Article	Art. 2(2)(t)(i), 51, 60	Art. 2(2)(t)(ii), 51, 60	Art. 27(1), 48(2), 2(2)(t), 51, 60
Legal conditions	App developer becomes a data holder if: (1) the app belongs to a relevant group of health-related products or services (2) it acts as a data controller (3) has consent or another legal basis to process data for qualified purposes	App developer becomes a data holder if: it can make non-personal health data available through control over the technical design of the product or related services, including registering, providing, restricting access to, or exchanging anonymised health data	Apps can self-label for EHR interoperability and transfer data to EHR with user consent. EHR operators become data holders if they fulfil the requirements of the Personal or Anonymous Health Data Pathway, independent of the app developer.
Practical examples	Health apps that collect health data and process it with authorisation (e.g., specific consent) beyond self-monitoring	Apps that collect, analyse, and sell anonymised health data	Digital therapeutic apps with EHR integration

Pathways and practical examples of how health data from health apps might be made available for secondary use under the EHDS Regulation by app developers for: (A) personal health data; (B) anonymous health data; or (C) independent of app developers via integration of health data in EHR. *EHDS*-R European Health Data Space Regulation, *EHR* Electronic Health Record.

Our study analyses the potential impact of the EHDS-R on app providers and their obligations to make user health data available for secondary use. However, some limitations apply. This analysis reflects the EHDS-R as adopted on 5 March 2025; later implementing acts, Commission guidance, and national rules may change obligations and our interpretation. We translated legal provisions into operational decision rules to code publicly available privacy policies; judgement was required for ambiguous terms such as research and innovation, so misclassification is possible. We did not audit data flows or verify claims with providers, and we could not assess whether data labelled as anonymised met robust standards rather than pseudonymisation. Our evaluation further relied on the assumption that consent obtained by the app provider is valid. However, it is well known that many consent practices, especially when the purpose is not clearly specified, are considered invalid. The sample comprised 100 apps from German, Irish, and Austrian app charts. The country selection was based on the author’s language proficiency to assess privacy policies and related information; this limits generalisability to the whole EU market, although many consumer apps are available EU-wide and duplicates were common in the country app store lists. The sample should therefore be interpreted as a market-facing sample of visible health apps in selected EU markets, not as a statistically representative sample of all health apps available in the EU. Additional selection bias may have been introduced by the sampling strategy and exclusion criteria. Apps with fewer than 10 reviews in both app stores were excluded because review counts served as a proxy for low user uptake. Apps with inaccessible privacy policies were also excluded because privacy policies were the primary data source for assessing health data processing and data holder qualification. However, these exclusions do not imply that such apps would fall outside the EHDS. In particular, apps without accessible privacy policies may still process relevant health data, but their lack of publicly available information prevented assessment in this study. Accordingly, the market-level quantification should be interpreted as an exploratory scenario estimate rather than a precise estimate of the number of EHDS-relevant health data holders in Europe. A further limitation of the assessment is that EU establishment status could not be reliably determined from app store listings or publicly available company information, particularly where app providers may operate through subsidiaries; territorial establishment was therefore not used as a criterion for data holder qualification.

App-generated health data will play an important role in our future healthcare system, complementing clinical data. The EHDS-R provides a framework for using this data transparently and securely for secondary purposes. While a significant number of app providers could face data-sharing obligations and impacts through the Personal and/or the Anonymous Health Data Pathway, it remains to be seen how this would be enforced in practice and how providers, especially those in the consumer health realm, would respond. For example, some may avoid research consent, and others may reduce data-sharing capabilities not to trigger respective pathways.

## Methods

We assessed 100 health apps using publicly available information from app stores, privacy policies, and product websites against EHDS health data holder criteria. The criteria were derived from the EHDS-R, and, where necessary, the GDPR, MDR, Artificial Intelligence Act, related EU legislation and relevant EU case law^1,6,15,16^.

### Regulatory assessment to develop EHDS health data holder criteria

According to Art. 60(1) and 51(1) of the EHDS-R, all *health data holders* are required to make *relevant electronic health data* available, upon request, to national HDABs, which act as central access points for the secondary use of health data. Whether and under what conditions data generated by health apps must be shared for secondary use depends on the interpretation of the two key concepts: relevant electronic health data and health data holder.

**Fig. 5:**
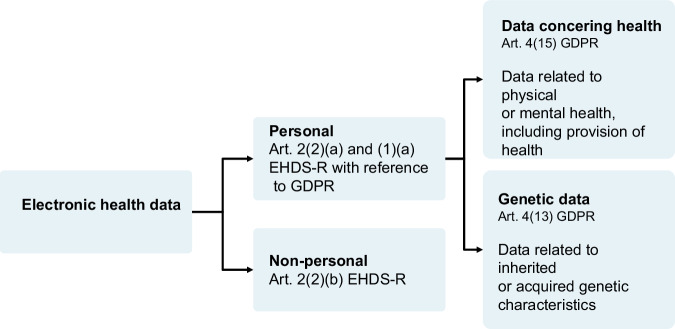
Definition of electronic health data in EHDS-R and their legal basis. Art. 51 EHDS-R further specifies the relevant categories of electronic health data for secondary use. The figure does not provide a general taxonomy of all GDPR personal data categories.

Art. 2(2)(c) EHDS-R defines electronic health data as ‘personal or non-personal electronic health data’. Personal health data is defined broadly, in line with GDPR, as personal data that can reveal an individual’s health status (Art. 2(1)(a) EHDS-R, Art. 4 No. 15 GDPR), whether directly or indirectly through collation and deduction^17^. Pseudonymised health data falls under the category of personal health data. Non-personal health data comprises all other health data, including anonymised data and data that never related to a data subject (Art. 2(2)(b) EHDS-R). App-generated health data fit within these categories as reinforced by Art. 51(1) EHDS-R, which explicitly lists data from MDs and wellness applications as relevant. Recent guidance publications on dataset description for the EHDS by TEHDAS2, an EU co-funded Joint Action supporting implementation of the EHDS, further clarify the scope of Article 51 categories and provide practical examples^18^. Fig. 5 summarises the definitions of electronic health data.

The second key concept, the health data holder, is more complex. In this study, the term *wellness application* follows the definition introduced by the EHDS-R (see Introduction). The term *health app(s)* is used as an umbrella concept encompassing any type of health app, irrespective of its EHDS-R or MD regulatory classification, intended purpose, or context of use. Hence, wellness applications are a subset of health apps.

Art. 2(2)(t) EHDS-R defines the health data holder by reference to entities ‘developing’ products or services or ‘developing or manufacturing’ wellness applications. It does not explicitly mention providers or operators. However, the purpose of the EHDS-R - to make data obtained through the operation of such applications available for secondary use (Recitals 52 and 56) - targets the entity that operates the app and controls the data, not necessarily the entity that wrote the source code. Therefore, in this legal assessment, we use the term ‘developer‘ in line with the wording of the regulation.

Whether an app developer qualifies as a health data holder under the EHDS-R, and is therefore required to make health data available for secondary use (Art. 61(1)), depends on the definition in Art. 2(2)(t), which differentiates between personal (Art. 2 (2)(t)(i)) and non-personal (anonymous) health data (Art. 2 (2)(t)(ii)), leading to distinct pathways (Table 3A and B). In both cases, the app developers must hold the data (Art. 60(3) EHDS-R). Apps where data remains on the user’s device and is inaccessible to the developer are excluded^12^.

To qualify as a health data holder under the Personal Health Data Pathway, a health app developer must meet three conditions (Table 3A). First, the app must either be intended for the health, healthcare or care sector, or qualify as a wellness application. This first criterion captures a broad range of health apps in commercial stores, so fine distinctions between groups are unnecessary, and an app developer’s grouping can remain open.

The second requirement is that the health app developer must qualify as a data controller (or joint controller). This is only the case if they decide on the purposes and means of processing health data (Art. 4 No. 7 GDPR), meaning they set the key processing parameters, such as why and how personal data are collected, stored, used, or shared^19^.

The third criterion requires that the health app developer has the right or the obligation to process the collected data for at least one of the EHDS-R listed purposes: (i) healthcare; (ii) care; (iii) public health; (iv) reimbursement; (v) scientific research (vi) innovation; (vii) policy making; (viii) official statistics; (ix) patient safety; or, (x) regulation. This criterion significantly narrows the group of health app developers that would qualify, and is likely to cause legal uncertainty. Under GDPR Articles. 6(1) and 9(2), such a right or obligation exists only under specific conditions, of which two are relevant here: either the app developer has obtained the user’s consent, or it is authorised or obligated by a qualified legal provision outside the EHDS-R (e.g., national law). Either of the two must directly relate to one of the purposes mentioned above (i-x). Consent to process data solely for the user’s own use, such as individual health analytics, falls outside this criterion, so those developers are not health data holders. However, the purposes themselves introduce ambiguity, as several terms lack clear definitions. For example, *research* or *innovation* is not defined, leaving room for interpretation and potential edge cases. In practice, consent to use data for usability or performance improvements may not qualify as innovation, but could AI model training? Likewise, academic literature generally assumes that consent for market research, which is commonly included in privacy policies, does not fall within the scope of research^20^.

In contrast to the Personal Health Data Pathway, the definition of data holder for non-personal (anonymous) health data is clearer (Table 3B). A health app developer qualifies as data holder if it can make non-personal electronic health data available through the control of the technical design of a product or service that enables registering, providing, restricting access to, or exchanging anonymised data (Art. 2(2)(t)(ii) EHDS-R).

The last route for making app-generated health data available for secondary use is through its voluntary integration into electronic health records (EHRs) via the EHR Interoperability Health Data Pathway (Table 3C). Manufacturers of both MD and wellness applications may claim interoperability with EHR systems, but they are subject to different requirements: MD manufacturers must prove compliance with the technical interoperability requirements for harmonised EHR software components specified in Section 2 of Annex II (Art. 27(1) EHDS-R), whereas wellness application manufacturers must issue a self-declared label confirming adherence to the common specifications and essential requirements (Art. 47(1) EHDS-R) with market surveillance authorities responsible for verifying this compliance (Art. 47(7) EHDS-R). A critical distinction exists in data transmission modalities: Art. 48(2) EHDS-R explicitly prohibits automatic data sharing for wellness applications and mandates that users must give consent and be able to select which specific data categories are to be entered into the EHR. While Art. 27 EHDS-R does not contain comparable specific provisions for MDs, any data transmission naturally requires a legal basis under Art. 6(1) GDPR. Following integration, the EHR operator, not the app developer, will become a data holder, provided they fulfil the requirements of the Personal or Anonymous Health Data Pathway. This EHR Interoperability Health Data Pathway is not part of the study, which focuses on the role of app developers as health data holders).

### App assessment

We retrieved the top 100 apps from the Google Play Store and Apple App Store in the categories “Health and Wellness” and “Medical,” including free and paid apps. App chart data, relating to April 15, 2025, was collected from the German, Irish and Austrian app store charts (date of collection April 28, 2025), using the mobile app data analysis platform from Foxdata Service^21^. These countries were selected because the authors had the language proficiency required to assess app store information, privacy policies, and linked public documents in German and English.

The inclusion criteria were on consumer-facing health apps used by individuals to manage personal health, well-being, and lifestyle. Eligible apps collected person-generated health data, either passively through usage or connected sensors (e.g., heart rate, activity), or actively through user input (e.g., diet, mood). Apps intended for healthcare professionals, clinical workflows, medical training, or pure educational purposes, without data-collection features, were excluded. Table 4 summarises all inclusion and exclusion criteria.

**Table 4:** Eligibility criteria for app screening

	Inclusion	Exclusion
App user group	Consumer-facing mobile health apps	Apps to be used by HCPs
App type	Apps for management of personal health and wellbeing that collect physical/ mental health data and lifestyle habits	Apps for supporting clinical workflows, medical training, and provider-provider communication Educational apps without data collection features Apps for pet health
Unit of analysis	Single mobile app	App bundles or app packs containing multiple individual apps
App stores	Google Play, Apple App Store	–
App store categories	Medical, Health, and Fitness	–
Pricing model	Free, paid	–
Country charts	Austria, Germany, Ireland	–
App language	English, German	–
Privacy Policy availability	Privacy Policy available in English or German and without purchasing app	–
App usage	10 or more reviews in at least one app store	–

Two reviewers screened country-specific app lists. To ensure consistency, both independently screened the top 100 apps from the German app stores and assigned a primary function. The app’s primary function was based on the dominant purpose presented in the app store description, acknowledging that many served multiple purposes. Discrepancies in screening and function labelling were resolved through discussion. One reviewer screened and labelled the remaining app lists. Function groups were then derived from these labels to guide later app selection (Supplementary Table 1). After screening, all lists were pooled and duplicates removed. To generate the 100-app sample for analysis, a stratified function group sampling approach was used to ensure that the final sample reflected the overall distribution of app function groups from the eligible app list while allowing greater representation of categories considered especially relevant in the context of the EHDS based on the value of the health data they may collect for secondary use. Patient portals, disease management apps, and sleep trackers were therefore oversampled because they were expected to be particularly informative for assessing the secondary use potential of app-generated data. To maintain the target sample size, categories such as fitness trackers and ePharmacy apps were sampled below their proportional share (Table 2). Within each function group, apps were randomly selected. Apps were excluded and replaced by an app of the same function group if their privacy policies were not accessible before purchasing or if they had fewer than 10 reviews in at least one app store.

Data extraction was conducted using an Excel sheet. One reviewer performed the data extraction after two reviewers had piloted and refined the data extraction sheet using a sample of 10 apps. App metadata and app characteristics, including provider country, app store ratings, function description, medical device status and target user group, were retrieved from the app store listing and product website. The privacy policies of health apps were the source of information on the collected health data types (including personal or anonymous health data status), health data storage and processing, legal basis, and controller status. Supplementary Table 2 presents the complete data extraction file. To protect the intellectual property of the app list provider, all apps were de-identified for reporting in this study.

All 100 apps were manually analysed by mapping the extracted information on health data processing (Supplementary Table 2) to the data holder qualification criteria derived from the EHDS-R (Table 3A and B). This mapping used the extracted information on health data type, app provider access or storage, controller or joint-controller status, stated processing purposes, legal basis, and statements on anonymous, anonymised, aggregated, or de-identified data. Apps were classified as qualifying, not qualifying or unclear for the Personal Health Data Pathway and the Anonymous Health Data Pathway. An “unclear” rating was assigned where the available Privacy Policy information was too broad or ambiguous to determine whether the relevant pathway criteria were met, including where policies referred to aggregated, de-identified, anonymous, or anonymised data without specifying whether these data included health data, whether they were derived from user health data, or whether they were anonymous rather than merely pseudonymised. The classification reflects data practices disclosed in privacy policies and related public sources; no technical verification of actual data flows, anonymisation methods, or re-identification risk was conducted. The final ratings for the two pathways for each app are included in the complete data extraction file (Supplementary Table 2).

## Supplementary information

Supplementary Information

## Data Availability

The app chart datasets analysed during the current study are not publicly available due to contractual restrictions with the app list provider, a commercial third party, but are available from the corresponding author on reasonable request. De-identified app data generated and analysed by the authors is provided in the Supplementary Information.

## Code availability

No code was generated during the current study.
