# Supplementary material for: Quantifying effects of the European Health Data Space on the app ecosystem and data access
Source: NPJ Digit Med. 2026 Jun 19;9:476. doi: 10.1038/s41746-026-02917-7 (PMC13282400; doi:10.1038/s41746-026-02917-7)
Supplement: Supplementary file 1 — Supplementary Information [file 41746_2026_2917_MOESM1_ESM.pdf]

Supplementary Results

Supplementary Table 1

	Function Group	Primary function
**1**	Fitness, Activity & Workout	Fitness and activity tracker; structured workout programmes
**2**	Disease Management & Therapy	Apps for managing health conditions and supporting therapy, e.g., chronic pain, smoking cessation
**3**	Nutrition & Calorie Counter	Apps for nutrition tracking and dietary support, e.g., calorie counters, diet planning
**4**	Sexual & Reproductive Health	Apps for sexual and reproductive health, e.g., menstrual tracking, pregnancy monitoring
**5**	Physiological Measurements	Apps for physiological measurements unrelated to disease or fitness, e.g., blood pressure, body temperature
**6**	Mental Health & Wellbeing	Apps for mental health and wellbeing, e.g., meditation, stress management
**7**	ePharmacy & ePrescription	Apps for e-pharmacy and e-prescription services, e.g., digital medication ordering, prescription management
**8**	Sleep Tracker	Apps for sleep tracking and analysis, e.g., monitoring sleep patterns, sleep quality
**9**	HCP Access & Communication	Apps for HCP access and HCP/patient communication, e.g., appointment booking, messaging
**10**	Speech & Cognitive Training	Apps to improve speech and cognitive capabilities, e.g., cognitive training games
**11**	Health Insurance Management	Apps for health insurance management, e.g., accessing insurance services, managing policy information
**12**	Diagnosis-support	Apps supporting the diagnosis of conditions and care recommendations, e.g., symptom checkers
**13**	Patient Health Record & Patient Portal	Apps supporting the storage of medical records for patients and exchange with providers
**14**	Device Control	Apps controlling hearing aids and recording hearing patterns
**15**	Hygiene, Cosmetic & Homoeopathy	Apps for oral and skin care, cosmetic and homoeopathic advice

Supplementary Table 1: App function groups derived from their primary functions. HCP Healthcare provider

Supplementary Table 2

	App characteristics				Medical Device Status	General information on Terms & Condition (T&C) and Privacy Policy (PP)					Information on health data processing from privacy policy								
**App ID**	**Main function**	**Function group**	**Brief app description**	**Target user group**	**Medical Device** (Risk class/No)	**T&C available** (Yes/No)	**T&C accessed** (In-app/ website)	**PP available** (Yes/No)	**PP accessed** (In-app/ website)	**Analysed language T&C/PP**	**Health data types processed**	**Health data processing and storage**	**Processing of personal health data for EHDS purposes**	**Edge case personal health data processing** (Analytics/Not mentioned)	**App provider is data controller for personal health data?**	**Legal basis for personal health data processing**	**Personal Health Data Pathway - Author's rating** (Yes/No/Unclear)	**Processing of anonymised health**	**Anonymous Health Data Pathway - Author's rating** (Yes/No/Unclear)
001	Appointment Booking	HCP Access & Communication	App allows publicly insured patients to book, manage, and find doctor and psychotherapist appointments.	Policyholders	No	No	Not available	Yes	Website	DE	Appoinment search and history	Mixed	Not mentioned	Not mentioned	Joint controller	Not applicable	No	Not mentioned	No
002	Nutrition & Calorie Counter	Nutrition & Calorie Counter	App provides a 30-day weight loss programm with guided workouts and diet plans, supporting calorie counting and progress tracking.	General public	No	Yes	In-app	Yes	Website	EN	Not specified but app collects weight, calories and exercises	User's device	Not mentioned	Not mentioned	No	Not applicable	No	Not mentioned	No
003	PHR & Patient Portal	PHR & Patient Portal	Electronic patient record app and for managing health insurance, submitting documents, and accessing insurance services.	Policyholders	No	Yes	Website	Yes	Website	DE	Various types including discharge letters, diagnoses, doctor's letters, findings, imaging examinations Emergency-relevant diagnoses, pre-existing conditions, operations and procedures, general medical data and more	App provider	Heathcare Reimbursment	Not mentioned	Yes	Not applicable	No	Not mentioned	No
004	Sleep Tracker	Sleep Tracker	App that automatically tracks and analyses sleep using data from a wearable device, offering insights into sleep stages, heart rate, and quality to help users understand and improve their sleep patterns.	General public	No	No	Not available	Yes	Website	DE	App analysis heart rate, active energy, sleep analysisfrom consumer health platform's database	User's device	Not mentioned	Not mentioned	No	Not applicable	No	Not mentioned	No
005	Health Insurance Management	Health Insurance Management	App for managing health insurance, submitting documents, and accessing insurance services.	Policyholders	No	No	Not available	Yes	Website	DE	Insurance status, submitted documents (e.g. invoices); information related to sick pay, medical aids, rehab services and others, reminder, digital health documents	App provider	Heathcare Reimbursment	Not mentioned	Yes	Not applicable	No	Not mentioned	No
006	Medication Tracking	Disease Management & Therapy	Contraceptive pill reminder with cycle tracking, symptom and mood logging and management of gynecologist appointments.	General public	No	Yes	Website	Yes	Website	DE	Not specified but app tracks medication intake, mood, symptoms and notes	App provider	Not mentioned	Not mentioned	Yes	Not applicable	No	Not mentioned	No
007	Workout	Fitness, Activity & Workout	App provides personalised workout and meal plans, mindfulness exercises, and tracking tools.	General public	No	Yes	Website	Yes	Website	DE	Age, sex, height, weight, desired weight, problem areas, data about training, preferences for meditation and breathing exercises, mental wellbeing, sleep patterns, type and duration of physical activities, physical condition, eating habits, water and food intake, food preferences, interactions with coaches<br><br>Data from connected wearables	App provider	Not mentioned	Not mentioned	Yes	Not applicable	No	Not mentioned	No
008	Physiological Measurements	Physiological Measurements	App for securely managing, tracking, and analysing a wide range of health data from over 30 devices, including options for sharing data with healthcare professionals.	Policyholders	IIa	Yes	Website	Yes	Website	DE	Height, sex, date of birth, pregnancy status, activity, puls, blood pressure, blood glucose, ECG, weight, medication, pulsoxy, sleep, temperature, fluid intake	Mixed	Not mentioned	Not mentioned	Joint controller	Not applicable	No	Not mentioned	No
009	Workout	Fitness, Activity	App to set and track fitness, nutrition and lifestyle goals	General public	No	Yes	Website	Yes	Website	EN	Health data not further specified, personal data generally<br>Photos	App provider	Not mentioned	Not mentioned	Yes	Not applicable	No	Not mentioned	No
010	Physiological Measurements	Physiological Measurements	App that allows users to manually log and track their blood pressure and blood glucose readings over time, providing visual charts and statistical summaries to monitor trends.	General public	No	No	Not available	Yes	In-app	DE	Weight, height, other information about health (including sexual activity) and related activities<br><br>Data import from other health apps possible (consent-based): weight, body temperature, calories burned, heart rate, steps/distance traveled, and other data about health	App provider	Not mentioned	Not mentioned	Yes	Not applicable	No	Not mentioned	No
011	Physiological Measurements	Physiological Measurements	App that allows users to track blood pressure, heart rate, and blood sugar, offering tools to organise this data and monitor trends over time.	General public	No	No	Not available	Yes	In-app	DE	Age, gender, weight, height, body temperature, menstrual cycle, symptoms, physical activities, location, other information about health and related activites	User's device	Not mentioned	Not mentioned	No	Not applicable	No	Not mentioned	No
012	Physiological Measurements	Physiological Measurements	App that allows users to manually record and monitor body temperature, symptoms, medications, and additional health parameters such as heart rate and weight.	General public	No	Yes	Website	Yes	Website	DE	Not specified but app collects body temperature, weight, and pulse	App provider	Not mentioned	Not mentioned	Yes	Not applicable	No	Not mentioned	No
013	Nutrition & Calorie Counter	Nutrition & Calorie Counter	App that tracks nutrition and calories using AI to estimate calorie and macronutrient content from meal photos, helping users log food intake and monitor diet goals efficiently.	General public	No	Yes	Website	Yes	Website	EN	Not specified but app collects photos of food and assesses calories	App provider	Not mentioned	Not mentioned	Yes	Not applicable	No	Not mentioned	No
014	Nutrition & Calorie Counter	Nutrition & Calorie Counter	Calorie counter and nutrition tracker app designed for individuals who want to monitor their diet, manage calorie intake, and reach weight loss or muscle gain goals.	General public	No	Yes	Website	Yes	Website	EN	Fitness and wellness data, health data from wearables integration	App provider	Research	Not mentioned	Yes	Consent	Unclear	Out of privacy policies's scope	Unclear

	App characteristics				Medical Device Status	General information on Terms & Condition (T&C) and Privacy Policy (PP)					Information on health data processing from privacy policy								
**App ID**	**Main function**	**Function group**	**Brief app description**	**Target user group**	**Medical Device** (Risk class/No)	**T&C available** (Yes/No)	**T&C accessed** (In-app/ website)	**PP available** (Yes/No)	**PP accessed** (In-app/ website)	**Analysed language T&C/PP**	**Health data types processed**	**Health data processing and storage**	**Processing of personal health data for EHDS purposes**	**Edge case personal health data processing** (Analytics/Not mentioned)	**App provider is data controller for personal health data?**	**Legal basis for personal health data processing**	**Personal Health Data Pathway - Author's rating** (Yes/No/Unclear)	**Processing of anonymised health**	**Anonymous Health Data Pathway - Author's rating** (Yes/No/Unclear)
015	Disease Management & Therapy	Disease Management & Therapy	App provides home-based access to a training plan created by user's therapist, including instructional videos and progress tracking. Users can rate exercises, document progress, and communicate feedback to their therapist. Usage of app only possible if healthcare provider uses software.	General public	No	Yes	Website	Yes	Website	DE	Health information entered by patients and healthcare provider, e.g., therapy plan, feedback<br><br>Data imported by connected device<br><br>Therapy related informtion	App provider	Healthcare	Not mentioned	Yes	Consent with download and app use	Yes	Not mentioned	No
016	Period, Fertility & Sexual Health	Sexual & Reproductive Health	Menstrual cycle tracker with personalised forecasting, symptom tracking, and support for pregnancy and menopause monitoring.	Women who want to track menstrual and reproductive health	I	Yes	Website	Yes	Website	DE	Cycle data and other health data (e.g., weight, body temperature, hair, sexual behaviour)<br><br>Data import from other health apps and wearables possible	App provider	Not mentioned	Not mentioned	Yes	Consent for research with anonymised data can be provided in app (DSGVO Art. 9)<br><br>Separate consent will be obtained for studies with clinical partners	No	Yes	Yes
017	PHR & Patient Portal	PHR & Patient Portal	Application that allows radiology patients to access, view, and share their medical imaging results and reports from healthcare providers.	General public	No	No	Not available	Yes	Website	DE	Health information uploaded by user (unspecified)<br>Diagnostic images and related information	App provider	Healthcare	Not mentioned	Yes (joint)	Consent with download and app use	Yes	Yes	Yes
018	Disease Management & Therapy	Disease Management & Therapy	Synchronises with glucose meter to provide personalised blood glucose monitoring, trend analysis, and diabetes self-management tools	Adults with diabetes	IIa	Yes	In-app	Yes	Website	DE	Blood glucose measures, Diabetes type, medication, insulin, food and exercise plan, healthcare provider information and clinical data, clinical reports	App provider	Research	Analytics	Yes	Consent with download and app use	Yes	Yes	Yes
019	Pregnancy & Parenting	Sexual & Reproductive Health	App that functions as a contraction timer, enabling users to log the start and end times of contractions and automatically calculate their duration and frequency for a summarised overview.	General public	No	No	Not available	Yes	Website	DE	Not specified	User's device	Not mentioned	Not mentioned	No	Not applicable	No	Not mentioned	No
020	Workout	Fitness, Activity & Workout	App guides users through a nine-week running program, helping them gradually progress from no activity to running a 5K, with audio cues, virtual coaches, workout tracking, music integration, GPS support, and community features.	General public	No	Yes	Website	Yes	Website	EN	Physical activity data	App provider	Not mentioned	Analytics	Yes	Not applicable	No	Might involve health data	Unclear
021	Nutrition & Calorie Counter	Nutrition & Calorie Counter	Nutrition tracking app for logging food, monitoring nutrients, and tracking health data such as weight and activity, with customizable goals, progress reports, and fitness device integration.	General public	No	Yes	Website	Yes	Website	DE	Nutrition and personal health related information (e.g. height, weight, nutrition, diet, exercise, health conditions, and targets)<br>Biometric information (e.g. keystrokes, behavioral or biological characteristics)<br>Demographic informatino: age, sex<br>Food photos plus related comments and corrections	App provider	Innovation	Analytics	Yes	Consent with download and app use	Unclear	Yes	Yes
022	Speech & Cognitive Training	Speech & Cognitive Training	App is a speech therapy programm based on Delayed Auditory Feedback.	General public	No	No	Not available	Yes	Website	EN	Not specified in PP but based on product description: Voice recordings	App provider	Not mentioned	Not mentioned	Yes	Not applicable	No	Out of privacy policies's scope	Unclear
023	Patient-HCP communication	HCP Access & Communication	App allows remote monitoring of patients' orthodontic treatment progress using regular intraoral photos. App is used with special accessories to take photos.	Patients undergoing orthodeontic treatments in collaboration with their healthcare provider	IIa	Yes	Not available	Yes	Website	EN	Not further specified in privacy policy but includes photos, treatment information and healthcare provider information	App provider	Healthcare Research	Analytics	Yes (joint)	Consent	Yes	Unclear if data is only aggregated or also anonymised	Unclear
024	Disease Management & Therapy	Disease Management & Therapy	App in connection with CGM to monitor blood glucose levels in real time	People with diabetes	IIb	Yes	Website	Yes	Website	DE	Demographic data (age, gender, disability status), video/audio/chat information; diabtes-related health information, appointments, genetic data, pregnancy-related information, medication, therapie details, and various other health information	App provider	Healthcare Research	Analytics	Yes	Yes	Yes	Might involve health data	Unclear
025	Disease Management & Therapy	Disease Management & Therapy	Therapeutic app designed to enhance fine motor and handwriting skills in children and adults, offering a series of hand and finger exercises that utilize the device's multi-touch interface to build strength, control, and dexterity.	General public	No	No	Not available	Yes	Website	DE	Personal data only, health data not specified	User's device	Not mentioned	Not mentioned	No	Not applicable	No	Not mentioned	No

	App characteristics				Medical Device Status	General information on Terms & Condition (T&C) and Privacy Policy (PP)					Information on health data processing from privacy policy								
**App ID**	**Main function**	**Function group**	**Brief app description**	**Target user group**	**Medical Device** (Risk class/No)	**T&C available** (Yes/No)	**T&C accessed** (In-app/ website)	**PP available** (Yes/No)	**PP accessed** (In-app/ website)	**Analysed language T&C/PP**	**Health data types processed**	**Health data processing and storage**	**Processing of personal health data for EHDS purposes**	**Edge case personal health data processing** (Analytics/Not mentioned)	**App provider is data controller for personal health data?**	**Legal basis for personal health data processing**	**Personal Health Data Pathway - Author's rating** (Yes/No/Unclear)	**Processing of anonymised health**	**Anonymous Health Data Pathway - Author's rating** (Yes/No/Unclear)
026	Disease Management & Therapy	Disease Management & Therapy	App is an electronic headache calendar capturing headache related information. It is also part of a national headache research project.	General public	No	No	Not available	Yes	Website	DE	Gender, birthday, height, weight, pregnancy related information, headache related information, medication etc.<br><br>If connected with treating healthcare professional, they will provide additional health data on the patinet (e.g., diagnosis, examination results tec)	App provider	Healthcare Research	Not mentioned	Yes	Consent with download and app use	Yes	Yes	Yes
027	ePharmacy	ePharmacy & ePrescription	Pharmacy app for online ordering of prescription and non-prescription medicines and pharmacy products, including digital redemption of e-prescriptions using a health insurance card.	General public	No	Yes	Website	Yes	Website	DE	Prescription data, medicine order history, health insurance number, communication data	App provider	Not mentioned	Not mentioned	Yes	Not applicable	No	Not mentioned	No
028	Sleep Tracker	Sleep Tracker	App generates adaptive soundscapes based on real-time inputs like heart rate for focus, relaxation and sleep.	General public	No	Yes	Website	Yes	Website	DE	Heart rate, physical activity	App provider	Not mentioned	Not mentioned	Yes	Not applicable	No	Might involve health data	Unclear
029	Nutrition & Calorie Counter	Nutrition & Calorie Counter	App for nutrition and symptom tracking with food, vitamin, and additive databases, plus health calculators and export.	General public	No	No	Not available	Yes	Website	DE	Nutrition data, weight, imported data from consumer health platform	User's device	Not mentioned	Not mentioned	No	Not applicable	No	Not mentioned	No
030	Diagnosis-support	Diagnosis-support	App detects and monitors irregular heart rhythms, such as atrial fibrillation, using a finger-on-camera (PPG sensor) measurement.	Adults at risk or with known cardiac arrhythmias, particularly atrial fibrillation (AF)	IIa	Yes	In-app	Yes	In-app	DE	Demographic informatinon, health status information e.g., medication, health measurements and annotations<br>Health data from connected devices	App provider	Healthcare	Not mentioned	Yes	Consent	Yes	Yes	Yes
031	Fitness & Activity Tracker	Fitness, Activity & Workout	App connects compatible smartwatches to smartphone, allowing users to monitor and record health metrics such as steps, sleep, heart rate, and blood oxygen, receive call and message notifications, and customise watch settings and reminders.	General public	No	No	Not available	Yes	Website	EN	Various physiological data: height, weight, gender, birthday, activity data sleep etc.<br>Device recorded data: Step count, exercise duration, sleep duration, impedance value, weight records, personal goals (exercise, weight)<br>Synced data from consumer health platforms	App provider	Research	Analytics/statistical analysis	Yes	Consent with download and app use	Yes	Yes	Yes
032	Workout	Fitness, Activity & Workout	App provides personalised workouts and progess tracking.	General public	No	Yes	Website	Yes	Website	EN	Birthdate, gender, height, weight, fitness activities<br><br>Data from connected wearables	App provider	Not mentioned	Not mentioned	Yes	Not applicable	No	Not mentioned	No
033	Period, Fertility & Sexual Health	Sexual & Reproductive Health	App for tracking periods, ovulation, cycle symptoms, and pregnancy, offering personalised insights, reminders, and expert content to support reproductive and menstrual health.	General public	No	Yes	Website	Yes	Website	EN	Weight, height, BMI, temperature, menstrual cycle dates, pregnancy related information, perimenopause and menopause symptoms, general well-being and health, symptoms, information on sex life, sleep	App provider	Research	Analytics	Yes	Consent	Yes	Yes	Yes
034	Fitness & Activity Tracker	Fitness, Activity & Workout	App for connecting smart device to track fitness, monitor health indicators, record workouts and manage health data.	General public	No	Yes	Website	Yes	Website	DE	Gender, date of birth, height, weight, activity level, sleep/wake times, activity and fitness data, workout data<br><br>Data from connected wearables	App provider	Not mentioned	Analytics	Yes	Not applicable	No	Not mentioned	No
035	ePharmacy	ePharmacy & ePrescription	Pharmacy app allows users to submit e-prescriptions digitally to local pharmacies, order and track medications for pickup or delivery, find healthcare providers nearby and access medication reminders	General public	No	Yes	Website	Yes	In-app	DE	Order information and history, gender, birthday<br>Medication tracking and reminder	Mixed	Not mentioned	Not mentioned	Joint controller	Not applicable	No	Not mentioned	No
036	Fitness & Activity Tracker	Fitness, Activity & Workout	Companion app for wearables supporting the tracking of exercise, step counts, sleep, heart rate and more.	General public	No	Yes	In-app	Yes	Website	DE	Height, weight, gender, age, activity data, heart rate, blood oxygen saturation, blood pressure, body temperature, ECG, respiration rate, body fat, muscle, protein, moisture, basal metabolism, internal organ fat, bone mass, body size, body age, calorie consumption, maximum oxygen uptake, training effect, training load, recovery time; mood, stress, emotional state; female health data<br>Data from connected wearables	App provider	Not mentioned	Not mentioned	Yes	Not applicable	No	Not mentioned	No
037	ePharmacy	ePharmacy & ePrescription	App allows users to shop for health and wellness products, receive personalised recommendations, earn rewards, explore expert wellness content, and locate nearby stores for convenient pickup or delivery.	General public	No	No	Not available	Yes	Website	IRL	Health care services: blood testing, laboratory & face scanning<br>Clinical professional services: phlebotomy, technology & data modelling services which provide biological age and wellness scores<br>Sleep, exercise	App provider	Research	Not mentioned	Yes	Consent	No	Not mentioned	No

	App characteristics				Medical Device Status	General information on Terms & Condition (T&C) and Privacy Policy (PP)					Information on health data processing from privacy policy								
**App ID**	**Main function**	**Function group**	**Brief app description**	**Target user group**	**Medical Device** (Risk class/No)	**T&C available** (Yes/No)	**T&C accessed** (In-app/ website)	**PP available** (Yes/No)	**PP accessed** (In-app/ website)	**Analysed language T&C/PP**	**Health data types processed**	**Health data processing and storage**	**Processing of personal health data for EHDS purposes**	**Edge case personal health data processing** (Analytics/Not mentioned)	**App provider is data controller for personal health data?**	**Legal basis for personal health data processing**	**Personal Health Data Pathway - Author's rating** (Yes/No/Unclear)	**Processing of anonymised health**	**Anonymous Health Data Pathway - Author's rating** (Yes/No/Unclear)
038	Mental Health & Wellbeing	Mental Health & Wellbeing	App supports mental health and emotional wellbeing with online therapy and meditation.	General public	No	Yes	Website	Yes	Website	DE	Gender, ethnicity, medical history, health-related details<br><br>Coach/therapy session related information	App provider	Not mentioned	Not mentioned	Yes	Not applicable	No	Out of privacy policies's scope	Unclear
039	Pregnancy & Parenting	Sexual & Reproductive Health	App that utilizes the device's microphone to capture a baby's heartbeat inside the womb.	General public	No	Yes	Website	Yes	Website	DE	Date of birth, gender, age	App provider	Not mentioned	Not mentioned	Yes	Not applicable	No	Not mentioned	No
040	Physiological Measurements	Physiological Measurements	App to measure and track heart rate.	General public	No	Yes	Website	Yes	Website	DE	Gender, Weight, Height, Body temperature, Menstrual cycle, Symptoms, other information about health (including sexual activities) and related activities<br><br>Data from connected wearables	App provider	Not mentioned	Not mentioned	Yes	Not applicable	No	Not mentioned	No
041	Mental Health & Wellbeing	Mental Health & Wellbeing	App that guides users through slow breathing exercises while measuring heart rate and variability using the phone's camera or a connected monitor, visualising each heartbeat, providing live feedback, integrating with consumer health platforms.	General public	No	Yes	Website	Yes	Website	DE	App locally stores camera data, heart rate, pulse, breath rate, breath curves, activity, wellbeing<br><br>Consumer health platform sync possible	User's device	Not mentioned	Not mentioned	No	Not applicable	No	Not mentioned	No
042	Workout	Fitness, Activity & Workout	Workout tracking app that lets users log strength training, create custom routines, monitor progress with stats and graphs, and engage with a community of athletes.	General public	No	Yes	Website	Yes	Website	DE	Movements, heartbeat, change in altitude or data about the surroundings	App provider	Not mentioned	Not mentioned	Yes	Not applicable	No	Not mentioned	No
043	Physiological Measurements	Physiological Measurements	App allows users to track, analyse, and share their blood pressure, pulse and weight, with interactive charts, color-coded status, and data export features.	General public	No	No	Not available	Yes	Website	EN	Body measurements & indexes; heart rate and other vital data	App provider	Not mentioned	Not mentioned	Yes	Not applicable	No	Not mentioned	No
044	Speech & Cognitive Training	Speech & Cognitive Training	Cognitive training app with games, exercises and personality tests.	General public	No	Yes	Website	Yes	Website	DE	Only personal data	App provider	Not mentioned	Not mentioned	Yes	Not applicable	No	Not mentioned	No
045	Appointment Booking	HCP Access & Communication	App to book doctor and therpaist appointments for on-site and telehealth consultations.	General public	No	Yes	In-app	Yes	Website	DE	Appointment-related information (time, date, type of medical examination, health insurance, medical history)<br>Profile information (e.g., medication, allergies)<br>Clinical data (provided by HCP)	App provider	Not mentioned	Not mentioned	Yes (joint)	Not applicable	No	Not mentioned	No
046	Disease Management & Therapy	Disease Management & Therapy	App providing a medically-based, personalized tinnitus therapy program including cognitive behavioral exercises, relaxation techniques, medical information, and progress tracking to help users manage and reduce tinnitus symptoms.	People with Tinitus	I	Yes	Website	Yes	Website	DE	Age, tinitus related information, progress information via survey data during therapy	App provider	Healthcare (app on prescription)	Not mentioned	Yes	Consent	Yes	Not mentioned	No
047	Pregnancy & Parenting	Sexual & Reproductive Health	App that enables users to record and track fetal movements, providing a log for kick sessions and allowing review of recorded activity.	General public	No	No	Not available	Yes	Website	DE	Not specified	User's device	Not mentioned	Not mentioned	No	Not applicable	No	Not mentioned	No
048	Disease Management & Therapy	Disease Management & Therapy	App for tracking headache occurrences, characteristics, and potential triggers. Users can log the duration, intensity, type of pain, medications taken, and other relevant factors.	General public	No	No	Not available	Yes	Website	DE	Personal data only, health data not specified	User's device	Not mentioned	Not mentioned	No	Not applicable	No	Not mentioned	No
049	Physiological Measurements	Physiological Measurements	App calculating body fat and lean body mass	General public	No	No	Not available	Yes	Website	DE	Health data (not specified)	User's device	Not mentioned	Not mentioned	No	Not applicable	No	Not mentioned	No
050	Fitness & Activity Tracker	Fitness, Activity & Workout	App that allows user to plan and track various exercise activties and share them with the app community	General public	No	Yes	Website	Yes	Website	DE	Activity and health information, photos<br>Health data from connected devices	App provider	Not mentioned	Not mentioned	Yes	Not mentioned	No	Yes	Yes
051	PHR & Patient Portal	PHR & Patient Portal	App provides patients in participating clinics with access to personalised therapy plans, treatment goals, questionnaires, and information related to their rehabilitation stay.	General public	No	Yes	Website	Yes	Website	DE	Phsical and mental health data, in-app surveys on health status, therapy plan	App provider	Research	Not mentioned	Yes	Consent with download and app use	Yes	Yes	Yes
052	Mental Health & Wellbeing	Mental Health & Wellbeing	App for self-discovery and transformation with tools for mindfulness, self-reflection, and emotional wellbeing.	General public	No	Yes	Website	Yes	Website	DE	Personal data not further specified for health data but may include date of birth; mood tracker, routine information and other user tracked data	App provider	Not mentioned	Analytics	Yes	Not applicable	No	Not mentioned	No

	App characteristics				Medical Device Status	General information on Terms & Condition (T&C) and Privacy Policy (PP)					Information on health data processing from privacy policy								
**App ID**	**Main function**	**Function group**	**Brief app description**	**Target user group**	**Medical Device** (Risk class/No)	**T&C available** (Yes/No)	**T&C accessed** (In-app/ website)	**PP available** (Yes/No)	**PP accessed** (In-app/ website)	**Analysed language T&C/PP**	**Health data types processed**	**Health data processing and storage**	**Processing of personal health data for EHDS purposes**	**Edge case personal health data processing** (Analytics/Not mentioned)	**App provider is data controller for personal health data?**	**Legal basis for personal health data processing**	**Personal Health Data Pathway - Author's rating** (Yes/No/Unclear)	**Processing of anonymised health**	**Anonymous Health Data Pathway - Author's rating** (Yes/No/Unclear)
053	Fitness & Activity Tracker	Fitness, Activity & Workout	App that converts physical activity, such as walking or running, into money by tracking steps and rewarding users in euros.	General public	No	Yes	Website	Yes	Website	DE	Steps, energy burned, distance walked, date of birth, weight, height, challenges	App provider	Not mentioned	Not mentioned	Yes	Not applicable	No	Might involve health data	Unclear
054	Mental Health & Wellbeing	Mental Health & Wellbeing	Self-care and habit tracker, enabling users to establish healthy habits. It features a daily routine planner and a mood tracker to help users manage their self-development and achieve personal goals.	General public	No	Yes	Website	Yes	Website	DE	Age, gender, physical characteristics (including height, weight, areas for improvement), fitness level, food preferences, food and exercise log, pictures taken and uploaded, AI instructions (text or picture used as AI prompt)<br><br>Data imported by other health apps or wearables	App provider	Not mentioned	Analytics	Yes	Not applicable	No	Yes	Yes
055	Health Passport	PHR & Patient Portal	App that creates a medical profile accessible from the lock screen, allowing users to share health details, emergency contacts, and location with first responders in case of emergency.	General public	No	Yes	Website	Yes	Website	EN	Personal data only, health data not specified but may include medical profile information (e.g., medical conditions, allergies, medications)	App provider	Not mentioned	Not mentioned	Yes	Not applicable	No	Not mentioned	No
056	Health Insurance Management	Health Insurance Management	App for managing health insurance, submitting documents, and accessing insurance services.	Policyholders	No	Not available	Website	Yes	In-app	DE	Social security data can be managed via the app, but no data is collected with the app	App provider	Healthcare Reimbursment	Not mentioned	No	Not applicable	No	Not mentioned	No
057	Fitness & Activity Tracker	Fitness, Activity & Workout	App for connecting smartwatch/smartband to track fitness, monitor health indicators, record workouts, manage health data, receive notifications, and enable payments and voice assistant features.	General public	No	Yes	In-app	Yes	Website	EN	Steps taken, standing activity and duration, exercise mode, cadence, distance, exercise time, elevation, heart rate, swim strokes, stroke rate, lap number, calories burned, sleep time and quality, stress level, gender, date of birth, height and weight, health plan information	App provider	Not mentioned	Not mentioned	Yes	Not applicable	No	Might involve health data	Unclear
058	PHR & Patient Portal	PHR & Patient Portal	App that provides access to lab results, appointments, medications, vaccination history, and other health records, along with secure messaging with healthcare providers and family account access, requiring an account with a participating healthcare provider to use these features.	General public	No	Yes	In-app	Yes	Website	DE	App can store any medical data provided by user/healthcare institution locally	User's device	Healthcare	Not mentioned	No	Not mentioned	No	Not mentioned	No
059	Nutrition & Calorie Counter	Nutrition & Calorie Counter	App for food and calorie logging as well as macronutrient tracking including a barcode scanner, recipe analysis, weight tracking, exercise logging, progress reports and nutrition goal monitoring.	General public	No	Yes	Website	Yes	Website	DE	Dietary habits; food, beverages and medications users consume; calorie counter; dietary restrictions; fitness levels, activity and goals; height, weight and BMI; Information on physiological conditions; photos, notes	App provider	Research	Not mentioned	Yes	Consent with download and app use	Unclear	Yes	Yes
060	Device Control	Device Control	App to control and personalise settings of hearing aids; remote device support, tracking of steps.	People with hearing loss	IIa	Yes	In-app	Yes	In-app	DE	Steps, activity level, hearing aid, device wearing time, time in different hearing environments, streaming time, heart frequency, distance, goals and wearing time, calories	App provider	Research	Not mentioned	Yes	Consent	Unclear	Not mentioned	No
061	Disease Management & Therapy	Disease Management & Therapy	App for logging and managing diabetes-related data such as blood glucose, insulin, carbs, meals, medications, and physical activity; supports integration with glucose meters and other health devices; suitable for users with type 1, type 2, or gestational diabetes.	People with diabetes (type 1, type 2, or gestational)	IIa	Yes	Website	Yes	Website	DE	Type and duration of activities (breakfast, office work, sports, etc.), food intake/meal/ingredients, tablet intake/injections, blood glucose measurements, notes/text, blood pressure, weight, HbA1c, ketones, steps, images/photos, medication, tags, points, imported values; sensor data such as start date/time, end date/time, time zone, sensor value, type; temporary basal rate, date; activated integrations; or coaching	App provider	Research	Not mentioned	Yes	Consent	Yes	Yes	Yes
062	Pregnancy & Parenting	Sexual & Reproductive Health	Baby sleep tracking app that helps parents monitor and manage their baby's sleep patterns.	General public	No	Yes	Website	Yes	Website	DE	Baby sleep data: sleep duration, wake times, naps, breastfeedings, diaper changes and pumping	App provider	Not mentioned	Not mentioned	Yes	Not applicable	No	Yes	Yes
063	Fitness & Activity Tracker	Fitness, Activity & Workout	Fitness app for tracking and analysing running activities, offering GPS tracking, training plans, and performance statistics.	General public	No	Yes	Website	Yes	Website	DE	Weight, height, body measurements, fitness and activity data	App provider	Not mentioned	Analytics	Yes	Not applicable	No	Not mentioned	No
064	Skin Care & Cosmetics	Hygiene, Cosmetic & Homeopathy	A barcode scanner app that decodes cosmetic ingredients and provides users with personalised recommednation for their skin situation.	General public	No	Yes	Website	Yes	Website	EN	Face photos, skin analysis information	App provider	Not mentioned	Not mentioned	Yes	Not applicable	No	Not mentioned	No

	App characteristics				Medical Device Status	General information on Terms & Condition (T&C) and Privacy Policy (PP)					Information on health data processing from privacy policy								
**App ID**	**Main function**	**Function group**	**Brief app description**	**Target user group**	**Medical Device** (Risk class/No)	**T&C available** (Yes/No)	**T&C accessed** (In-app/ website)	**PP available** (Yes/No)	**PP accessed** (In-app/ website)	**Analysed language T&C/PP**	**Health data types processed**	**Health data processing and storage**	**Processing of personal health data for EHDS purposes**	**Edge case personal health data processing** (Analytics/Not mentioned)	**App provider is data controller for personal health data?**	**Legal basis for personal health data processing**	**Personal Health Data Pathway - Author's rating** (Yes/No/Unclear)	**Processing of anonymised health**	**Anonymous Health Data Pathway - Author's rating** (Yes/No/Unclear)
065	Disease Management & Therapy	Disease Management & Therapy	App offers digital therapy for adipositas and supports patients to reduce weight and change habits.	People with adipositas in Germany	I	Yes	Website	Yes	Website	DE	Body weight, BMI, waist circumference, blood pressure, blood sugar, mood, activity levels, and eating habits, workout/fitness activities (steps, calories burned, type and duration of activity), data on lifestyle, symptoms, and progress in therapy<br><br>Health data from connected devices	App provider	Healthcare (app on prescription)<br>Research	Not mentioned	Yes	Consent	Yes	Yes	Yes
066	Period, Fertility & Sexual Health	Sexual & Reproductive Health	App used for contraception or pregnancy planning that calculates ovulation, fertile window, and period based on body signals such as temperature, allowing users to log symptoms and export cycle data for healthcare professionals.	People planning for contraception or pregnancy	IIb	Yes	Website	Yes	Website	DE	Cycle tracking data, additional body health signals, pregnancy data, and other health and symptom logs	App provider	Not mentioned	Not mentioned	Yes	Not applicable	No	Not mentioned	No
067	Physiological Measurements	Physiological Measurements	App measures interpupillary distance.	General public	No	Yes	Website	Yes	Website	EN	Personal data generally covered	User's device	Not mentioned	Not mentioned	No	Not applicable	No	Not mentioned	No
068	Disease Management & Therapy	Disease Management & Therapy	Asthma diary app for tracking lung function.	General public	No	Yes	Website	Yes	Website	DE	Birthday, age, logged data (not specified)	App provider	Not mentioned	Analytics (health data usage not specified)	Yes	Not applicable	No	Not mentioned	No
069	Disease Management & Therapy	Disease Management & Therapy	App for tracking and assessing Peak Expiratory Flow (PEF) for asthma management that allows users to log, view, and report their PEF readings, track medication effects, and create reports to support self-monitoring and communication with healthcare professionals.	General public	No	No	Not available	Yes	Website	EN	Personal data only, health data not specified	App provider	Not mentioned	Not mentioned	Yes	Not applicable	No	Not mentioned	No
070	Workout	Fitness, Activity & Workout	App allows users to create and follow personalised training plans, track workout progress, access an exercise database, plan meals, and sync data with health platforms.	General public	No	Yes	Website	Yes	Website	DE	Height, weight, BMI, age, biological sex, preferences, completed workouts and runs, steps, calories burned, location data, other information user voluntarily provides, calory intake, used recipes and products, user generated content such as pictures or posts	App provider	Not mentioned	Not mentioned	Yes	Not applicable	No	Not mentioned	No
071	Period, Fertility & Sexual Health	Sexual & Reproductive Health	App enables users to track their menstrual cycles, ovulation, fertility windows, and related health symptoms.	General public	No	Yes	In-app	Yes	Website	EN	Only stored on devcie: Menstrual cycle, period dates and lenght, symptoms and moods, sexual activity, contraception, medication, temperature and weight measurements, personal notes	User's device	Not mentioned	Not mentioned	No	Not applicable	No	Not mentioned	No
072	Workout	Fitness, Activity & Workout	App offers guided wall Pilates workouts for women and features personalised plans, video instructions, and progress tracking.	General public	No	No	Not available	Yes	Website	EN	Generic list of health data not tailored to app	App provider	Not mentioned	Not mentioned	Yes	Not applicable	No	Not mentioned	No
073	Disease Management & Therapy	Disease Management & Therapy	App that supports nutrition, exercise, and mental health for people with breast cancer, offering personalized daily goals, educational content, mindfulness training, and practical tools to improve quality of life and manage therapy side effects.	Patients with breast cancer diagnosis and mild depressive symptoms	I	Yes	Website	Yes	Website	DE	Patient demographics, diagnosis (cancer status, treatment stage), therapy related information, symptom tracking, side effect tracking, nutrition and dietary habits, physical activity and exercise routines, mental health information, uploaded medical findings/documents	App provider	Healthcare (app on prescription)	Not mentioned	Yes	Consent with download and app use	Yes	Not mentioned	No
074	Pregnancy & Parenting	Sexual & Reproductive Health	App that provides pregnancy tracking, daily content, 3D baby development models, symptom tracking, and weekly advice with checklists and planning tools for expectant parents.	General public	No	Yes	Website	Yes	Website	DE	Pregnancy related information (due date, day/week of pregnancy, loss of pregnancy, relationship to baby)<br><br>Consumer health platform integration possible	App provider	Not mentioned	Not mentioned	Yes	Not applicable	No	Yes	Yes
075	Sleep Tracker	Sleep Tracker	App that records and analyses night-time sounds like snoring and sleep talking, offering automatic sound detection, audio playback, and noise-level graphs to monitor sleep patterns.	General public	No	Yes	Website	Yes	Website	DE	Consumer health platform data	App provider	Not mentioned	Not mentioned	Yes	Not applicable	No	Not mentioned	No
076	Addiction Support	Disease Management & Therapy	Provides support for quitting smoking by tracking progress, offering motivational tools, and giving tips to help users remain smoke-free.	General public	No	Yes	Website	Yes	Website	DE	Non-smoking days, not smoked cigarettes	User's device	Not mentioned	Not mentioned	No	Not applicable	No	Not mentioned	No

	App characteristics				Medical Device Status	General information on Terms & Condition (T&C) and Privacy Policy (PP)					Information on health data processing from privacy policy								
**App ID**	**Main function**	**Function group**	**Brief app description**	**Target user group**	**Medical Device** (Risk class/No)	**T&C available** (Yes/No)	**T&C accessed** (In-app/ website)	**PP available** (Yes/No)	**PP accessed** (In-app/ website)	**Analysed language T&C/PP**	**Health data types processed**	**Health data processing and storage**	**Processing of personal health data for EHDS purposes**	**Edge case personal health data processing** (Analytics/Not mentioned)	**App provider is data controller for personal health data?**	**Legal basis for personal health data processing**	**Personal Health Data Pathway - Author's rating** (Yes/No/Unclear)	**Processing of anonymised health**	**Anonymous Health Data Pathway - Author's rating** (Yes/No/Unclear)
077	Addiction Support	Disease Management & Therapy	App guides users through the process of quitting smoking by offering motivation, progress tracking, customisable quit methods and supportive features like health tracking, savings goals, craving analysis, tips, games and peer challenges.	General public	No	Yes	Website	Yes	Website	DE	Data on smoking behaviour, sex, age	App provider	Not mentioned	Analytics (health data usage not specified)	Yes	Not applicable	No	Not mentioned	No
078	Mental Health & Wellbeing	Mental Health & Wellbeing	App that provides a customisable AI chatbot companion for conversations, emotional support, mood tracking, and mental well-being, helping users improve self-reflection, manage anxiety, and build coping skills through interactive chat.	General public	No	Yes	Website	Yes	Website	EN	Messages and content (including video, voice and text), interests and conversation preferences (e.g., topics), biometric information (voice, head movement, face	Mixed	Not mentioned	Not mentioned	Joint controller	Not applicable	No	Might involve health data (chat conversations)	Unclear
079	ePharmacy	ePharmacy & ePrescription	Pharmacy app for ordering medications, beauty, and personal care products with home delivery. Allows users to redeem e-prescriptions via their health insurance card.	General public	No	Yes	Website	Yes	Website	DE	Purchased products, order history	App provider	Not mentioned	Not mentioned	Yes	Not applicable	No	Not mentioned	No
080	Sleep Tracker	Sleep Tracker	App that uses an AI-driven algorithm to analyse sleep sounds, track sleep cycles and snoring patterns, and includes a smart alarm that wakes users during their lightest sleep stage.	General public	No	Yes	Website	Yes	Website	DE	Age, gender, physical characteristics , fitness level, food preferences, food and exercise log, pictures taken and uploaded, AI instructions (text or picture used as AI prompt)<br><br>Imported data from other health apps	App provider	Not mentioned	Analytics	Yes	Not applicable	No	Not mentioned	No
081	Health & Fitness	Fitness, Activity & Workout	App provides yoga exercise videos and users can personalise their routines.	General public	No	Yes	Website	Yes	Website	EN	Weight, age, gender	App provider	Not mentioned	Not mentioned	Yes	Not applicable	No	Might involve health data	Unclear
082	Diagnosis-support	Diagnosis-support	Analyses photos of skin spots or moles to assess the risk of skin cancer and advises if medical attention is recommended.	Adults who want to monitor their skin for signs of skin cancer and receive guidance on when to seek a professional evaluation	I	Yes	Website	Yes	Website	DE	Birthday, age, sex, information on skin type and riskprofile, skin images, annotations,<br>histopathological report uploaded by user	App provider	Research	Not mentioned	Yes	Yes if app is used in clinical study<br>No additional consent collected for internal research and aggregated/anonymised data usage with external research partner	Yes	Yes	Yes
083	Sleep Tracker	Sleep Tracker	Sleep schedule app that helps users build regular sleep habits by rewarding consistent bedtimes and wake-up times with virtual city-building, featuring challenges with family and friends, reminders, progress tracking, and wearable integration.	General public	No	Yes	Website	Yes	Website	EN	Sleep activity<br>Imported data from other health apps	App provider	Not mentioned	Not mentioned	Yes	Not applicable	No	Not mentioned	No
084	Addiction Support	Disease Management & Therapy	App to support quitting drinking by tracking progress, offering motivational tools and games.	General public	No	No	Not available	Yes	In-app	DE	Alcohol consumption related information, age, gender, fitness related information	User's device	Not mentioned	Not mentioned	No	Not applicable	No	Not mentioned	No
085	Disease Management & Therapy	Disease Management & Therapy	Provides pelvic floor muscle exercise plans, reminders, and progress tracking to help women manage and improve pelvic health	Women with pelvic floor dysfunction or as prevention	I	Yes	Website	Yes	Website	EN	Medical history, health status, exercises, patient reported outcomes, diary results, flares/episodes/symptoms, test results, diagnosis, medication	App provider	Healthcare Research	Not mentioned	Yes	Not mentioned, vague description that "most of the time" anonymised data is used	Yes	Yes	Yes
086	Workout	Fitness, Activity & Workout	App that offers guided stretching exercises through videos and customisable workout plans, including features like interval timers and workout scheduling.	General public	No	Yes	Website	Yes	Website	DE	Uploades training programs, workout statistics, calories burned, other fitness information, if account is connected to third party applications health-related data synced with the services from the third-party apps	App provider	Not mentioned	Not mentioned	Yes	Not applicable	No	Not mentioned	No
087	Fitness & Activity Tracker	Fitness, Activity & Workout	Offline step counter measuring steps and calories as well as walking distance and duration.	General public	No	No	Not available	Yes	In-app	DE	Step and activity data, age, gender, weight, height	Mixed	Not mentioned	Not mentioned	Joint controller	Not applicable	No	Not mentioned	No
088	Fitness & Activity Tracker	Fitness, Activity & Workout	App for habit tracking that enables users to set daily tasks, receive reminders, monitor completion, and view progress statistics to build routines, with integration into a consumer health platform.	General public	No	No	Not available	Yes	Website	DE	Activity, mindfulness, nutrition, sleep, body measurements, vitals, heart	User's device	Not mentioned	Not mentioned	No	Not applicable	No	Not mentioned	No
089	Fitness & Activity Tracker	Fitness, Activity & Workout	App that offers exercise routines and general tips intended to support natural growth.	General public	No	Yes	Website	Yes	Website	DE	Size, weight, gender, age, ethnicity, parent's size, shoe size, training routines, sleep habits	App provider	Not mentioned	Not mentioned	Yes	Not applicable	No	Not mentioned	No

	App characteristics				Medical Device Status	General information on Terms & Condition (T&C) and Privacy Policy (PP)					Information on health data processing from privacy policy								
**App ID**	**Main function**	**Function group**	**Brief app description**	**Target user group**	**Medical Device** (Risk class/No)	**T&C available** (Yes/No)	**T&C accessed** (In-app/ website)	**PP available** (Yes/No)	**PP accessed** (In-app/ website)	**Analysed language T&C/PP**	**Health data types processed**	**Health data processing and storage**	**Processing of personal health data for EHDS purposes**	**Edge case personal health data processing** (Analytics/Not mentioned)	**App provider is data controller for personal health data?**	**Legal basis for personal health data processing**	**Personal Health Data Pathway - Author's rating** (Yes/No/Unclear)	**Processing of anonymised health**	**Anonymous Health Data Pathway - Author's rating** (Yes/No/Unclear)
090	Workout	Fitness, Activity & Workout	App that enables users to follow personalised training programmes, log workouts, connect with equipment for automatic data tracking, monitor performance metrics over time, and access video-guided exercises.	General public	No	Yes	Website	Yes	Website	DE	Gender, weight, height, body composition, date of birth, training and activity data, data collected by gym machines,<br>Photos (training results), audio, contacts and calendar information (to program a training fitness programme)<br><br>Data from connected devices devices	App provider	Not mentioned	Not mentioned	Yes	Not applicable	No	Might involve health data	Unclear
091	Nutrition & Calorie Counter	Nutrition & Calorie Counter	App that provides information on optimal nutrition based on blood type, allowing users to search for specific foods and create shopping lists.	General public	No	No	Not available	Yes	Website	DE	Not specified	User's device	Not mentioned	Not mentioned	No	Not applicable	No	Not mentioned	No
092	Disease Management & Therapy	Disease Management & Therapy	Allergy support app by health insurance for individuals with pollen allergies providing regional pollen forecasts, symptom tracking, and information about allergens, symptom and medication tracking.	General public	No	No	Not available	Yes	Website	DE	Symptom diary entries, medication used, allergens selected for tracking, data from self-test	Mixed	Not mentioned	Not mentioned	Joint controller	Not applicable	No	Yes	Yes
093	Mental Health & Wellbeing	Mental Health & Wellbeing	App supports biofeedback meditation with real-time breath and pulse detection, offering breathing exercises and visualizations.	General public	No	No	Not available	Yes	Website	DE	Respiratory pattern, respiratory rate, abdominal respiration/breath curve, heart rate, heart rate variability, mindful minutes	Mixed	Not mentioned	Not mentioned	Joint controller	Not applicable	No	Yes	Yes
094	Speech & Cogni	Speech & Cogni	App for voice pitch monitoring and recording with features for real-time analysis.	General public, singer, people with Parkinson's and transgender	No	No	Not available	Yes	Website	EN	None	User's device	Not mentioned	Not mentioned	No	Not mentioned	No	Not mentioned	No
095	Disease Management & Therapy	Disease Management & Therapy	App on prescription that offers exercise-based therapy plans, tracking, and daily guidance to help users manage and reduce back pain	Adults with back pain	I	Yes	Website	Yes	Website	DE	Indication, symptom-related information, medication, reminders for training, training-related information, daily step counts, adherence, progress questionnaires and reports, gender	App provider	Healthcare (app on prescription)	Not mentioned	Yes	Not mentioned	Yes	Not mentioned	No
096	Nutrition & Calorie Counter	Nutrition & Calorie Counter	App calculates personal daily water needs, tracks water intake, and provides reminders to help users maintain healthy drinking habits.	General public	No	Yes	Website	Yes	Website	DE	Hydration data, hydration goals	User's device	Not mentioned	Not mentioned	No	Not applicable	No	Not mentioned	No
097	Fitness & Activity Tracker	Fitness, Activity & Workout	App that provides personalised recommendations on sleep, exercise, stress, and overall health based on physiological measurements collected by a wearable device.	General public	No	Yes	Website	Yes	Website	DE	Heart rate, heart rate variability, respiratory rate, skin temperature and blood oxygen saturation; acceleration; metadata about workouts and sleep; the type of physical activity users engage in and the duration of activity; data reflecting exertion and recovery; users physiological profile, including birthday, gender identity, weight, height, fitness/athlete level; and details user chooses to provide about habits, diet, medications and female health tracking; communication with AI Coach	App provider	Research	Analytics	Yes	Not mentioned	Yes	Yes	Yes
098	Fitness & Activity Tracker	Fitness, Activity & Workout	App for securely managing, tracking, and analysing a wide range of health data from over 30 devices, including options for sharing data with healthcare professionals.	General public	No	No	Not available	Yes	Website	DE	Workout information (stored only on users phone)	User's device	Not mentioned	Not mentioned	No	Not applicable	No	Not mentioned	No
099	Nutrition & Calorie Counter	Nutrition & Calorie Counter	App for tracking calories, intermittent fasting and nutrition, offering a food diary, fasting plans, recipes, activity tracking, and personalised meal plans to support healthy weight management and muscle building.	General public	No	Yes	Website	Yes	Website	DE	Age, date of birth, gender, height, diet, health goals, activities, calories, steps, weight, blood pressure, fasting time, water intake,	App provider	Not mentioned	Not mentioned	Yes	Not applicable	No	Not mentioned	No
100	Workout	Fitness, Activity & Workout	App that offers yoga routines and targeted exercises for all levels to help users lose weight, tone their body, and improve flexibility, posture, and mental well-being, with features for tracking calories burned and progress.	General public	No	No	Not available	Yes	Website	EN	Generic list of health data not tailored to app	App provider	Not mentioned	Not mentioned	Yes	Not applicable	No	Not mentioned	No

Supplementary Table 2 Data extraction file

Supplementary Table 3

	Total	MD	Non-MD	Personal Health Data Pathway		Anonymous Health Data Pathway	
				Yes	Unclear	Yes	Unclear
Fitness, Activity & Workout	23	0	23	2	0	3	5
Disease Management & Therapy	19	8	11	10	0	6	1
Nutrition & Calorie Counter	9	0	9	0	3	2	1
Sexual & Reproductive Health	9	2	7	1	0	4	0
Physiological Measurements	8	1	7	0	0	0	0
Mental Health & Wellbeing	6	0	6	0	0	2	2
ePharmacy & ePrescription	4	0	4	0	0	0	0
Sleep Tracker	5	0	5	0	0	0	1
HCP Access & Communication	3	1	2	1	0	0	1
Speech & Cognitive Training	3	0	3	0	0	0	1
Health Insurance Management	2	0	2	0	0	0	0
Diagnosis-support	2	2	0	2	0	2	0
PHR & Patient Portal	5	0	5	2	0	2	0
Device Control	1	1	0	0	1	0	0
Hygiene, Cosmetic & Homeopathy	1	0	1	0	0	0	0
	**100**	**15**	**85**	**18**	**4**	**21**	**12**

Supplementary Table 3: Evaluation results of app qualification as data holder for the Personal and Anonymous Health Data Pathway qualification health apps per app function group. The numbers of qualifying apps are highlighted in green.
